# Distribution and prognosis of uncommon metastases from non-small cell lung cancer

**DOI:** 10.1186/s12885-016-2169-5

**Published:** 2016-02-24

**Authors:** Fei-Yu Niu, Qing Zhou, Jin-Ji Yang, Wen-Zhao Zhong, Zhi-Hong Chen, Wei Deng, Yan-Yan He, Hua-Jun Chen, Zhu Zeng, E-E Ke, Ning Zhao, Na Zhang, Hui-Wen Sun, Qiu-Yi Zhang, Zhi Xie, Xu-Chao Zhang, Yi-Long Wu

**Affiliations:** Southern Medical University, Guangzhou, China; Guangdong Lung Cancer Institute, Guangdong General Hospital & Guangdong Academy of Medical Sciences, Guangzhou, China

**Keywords:** NSCLC, Uncommon metastases, Prognosis, Local treatment

## Abstract

**Background:**

According to the literature and our experience, the most common sites of non-small cell lung cancer (NSCLC) metastases include the brain, bone, liver, adrenal glands, contralateral lung and distant lymph nodes. Metastases to other organs are relatively rare. There have been numerous case reports and a few small case series of uncommon metastases derived from NSCLC.

**Methods:**

We defined all organs except the common metastatic sites mentioned above as uncommon sites of metastasis. Patients with uncommon metastases among 2,872 consecutive NSCLC patients with stage IV disease at the Guangdong Lung Cancer Institute (GLCI) from 2006 to 2012 were included in this study. The diagnosis of uncommon metastases was based on pathology or imaging studies.

**Results:**

Uncommon metastases were diagnosed in 193 cases at anatomical sites such as the soft tissue, kidney, pancreas, spleen, peritoneum, intestine, bone marrow, eye, ovary, thyroid, heart, breast, tonsil and nasal cavity. Uncommon metastases were identified as independent poor prognostic factors through a multivariate analysis with a HR (hazard ratio) of 1.29 [95 % confidence interval (CI) 1.09–1.52, *P <* 0.01]. Those patients who received systemic therapy plus local treatment had a better survival rate than did those who received systemic therapy only (*P* < 0.01); all patients received best supportive care.

**Conclusions:**

Metastases to the above mentioned sites are infrequent. The presentation of uncommon metastases tends to indicate a poor outcome, and selected patients may benefit from local treatment.

## Background

Approximately 50 % of lung cancer cases are metastatic at diagnosis [1]. The major sites of non-small cell lung cancer (NSCLC) metastases include the brain (47 %), bone (36 %), liver (22 %), adrenal glands (15 %), thoracic cavity (11 %) and distant lymph nodes (10 %) [2, 3]. All other organs metastases are very rare and general less than 5 %. So we could define them as uncommon metastases. To our knowledge only some case reports were presented and there were limit information available on uncommon metastases of NSCLC in the literatures [4–12]. Thus, no systematic body of knowledge on the epidemiology, diagnosis, or treatment of such metastases is available. Misdiagnosis is common because it is difficult to distinguish uncommon metastases from primary malignancies. The treatment is often controversy when uncommon metastasis is solitary. Local therapy is always considered in such settings, although uncommon metastases are grouped as M1b, stage IV based on lung cancer staging system [13]. Herein, we review all cases with uncommon metastases identified at the Guangdong Lung Cancer Institute (GLCI) between 2006 and 2012, and we report the epidemiological characteristics, diagnosis, treatment and prognosis of the patients. Our purpose was to evaluate survival outcomes, to define predictors of survival, and to provide information to clinicians on how to treat uncommon metastases derived from NSCLC.

## Patients and methods

The definition of uncommon metastases is metastatic sites exclusive of the brain, bone, liver, adrenal glands, thoracic cavity and distant lymph nodes. All patients diagnosed with uncommon metastases from 2,872 consecutive NSCLC patients with stage IV disease, at the initial presentation or during follow-up at the GLCI from 2006 to 2012 were included in this study, which was approved by the ethics committee of Guangdong General Hospital. All patients provided written informed consent for participation were included in the study. Otherwise they were not included. The diagnosis of soft tissue metastases was based on pathology, imaging results, or clinical manifestations. Metastases located in the skeletal muscle/subcutaneous/cutaneous tissues were included,however, those in important lymphatic drainage areas (e.g., the groin and axilla) were excluded unless they were pathologically diagnosed as soft tissue metastases. Metastases located in lymphatic regions are more likely to be metastases in lymph nodes rather than soft tissue. The diagnosis of other uncommon metastatic sites (e.g., the kidneys and pancreas) depended mostly on imaging studies, including computed tomography (CT), magnetic resonance imaging (MRI), and positron emission tomography/computed tomography (PET/CT). The diagnosis of metastases to those organs was confirmed by two independent radiologists and a physician.

We wonder whether patients with uncommon metastases diagnosed at different time would experience different prognoses. Thus, we defined two clinical situations: synchronous and metachronous diagnoses. Synchronous metastases were defined as clinically and/or radiologically uncommon metastases identified at the time of lung cancer diagnosis. Metachronous metastases were defined as uncommon metastases diagnosed after the initial diagnosis of primary lung cancer.

Chi-square or Fisher’s exact tests were used to compare qualitative data. Nonparametric tests were used to analyze the quantitative data. Overall survival (OS) was estimated using the Kaplan-Meier method, and the difference in survival between the subgroups was compared using a log-rank test. To estimate the risk of OS in the cohort of 2,872 patients, the group (common or uncommon metastasis), age, gender, cigarette smoking history, ECOG PS, pathology and systematic treatment or not were used as covariates in a multivariate Cox regression model. All analyses were performed using the SPSS 17.0 software program. All statistical tests were two-sided, and *P* < 0.05 was deemed to indicate statistical significance.

## Results

Overall, 193 cases (6.7 %) were identified as having uncommon metastases among 2,872 consecutive NSCLC cases from 2006 to 2012. Sixteen cases had more than one uncommon metastatic organ.

### Clinical features

The clinical characteristics of cases with common and uncommon metastases are shown in Table 1. Compared with the common metastasis group, the patients in the uncommon metastasis group were more likely have metachronous metastases (*P* < 0.01) and were more likely to be male (*P* = 0.02). Other clinical factors such as age, smoking status, ECOG PS, histology and treatment were balanced between the two groups.

**Table 1:** Clinical characteristics of cases with uncommon and common metastasis

	Uncommon metastases		Common metastases		*P*
	n	%	n	%	
Age (years)					0.36
Median (range)	58 (20–85)		59 (17–89)		
Gender					0.02
Male	137	71.0	1673	62.4	
Female	56	29.0	1006	37.6	
Smoking status					0.08
Smoker	99	51.3	1197	44.7	
Never smoker	94	48.7	1482	55.3	
ECOG PS					0.96
<2	175	90.7	2423	90.4	
=2	12	6.2	178	6.6	
>2	6	3.1	78	2.9	
Diagnosis time					<0.01
At disease course	82	43.0	415	15.5	
At intial	111	57.0	2264	84.5	
Histology					0.41
Adenocarcinoma	151	78.2	2178	81.3	
Squamous carcinoma	26	13.5	339	12.7	
Others^a^	16	9.0	162	6.0	
Treatment					
>2 lines	35	18.1	410	15.3	0.49
≤2 lines	122	63.2	1795	67.0	
BSC	36	18.7	474	17.7	
TKIs or not					0.06
Yes	56	29.0	959	35.8	
No	137	71.0	1720	64.2	

*Abbreviations*: *ECOG PS* Eastern Cooperative Oncology Group performance status

^a^Other types of histology except adenocarcinomas and squamous carcinoma

**Fig. 1:**
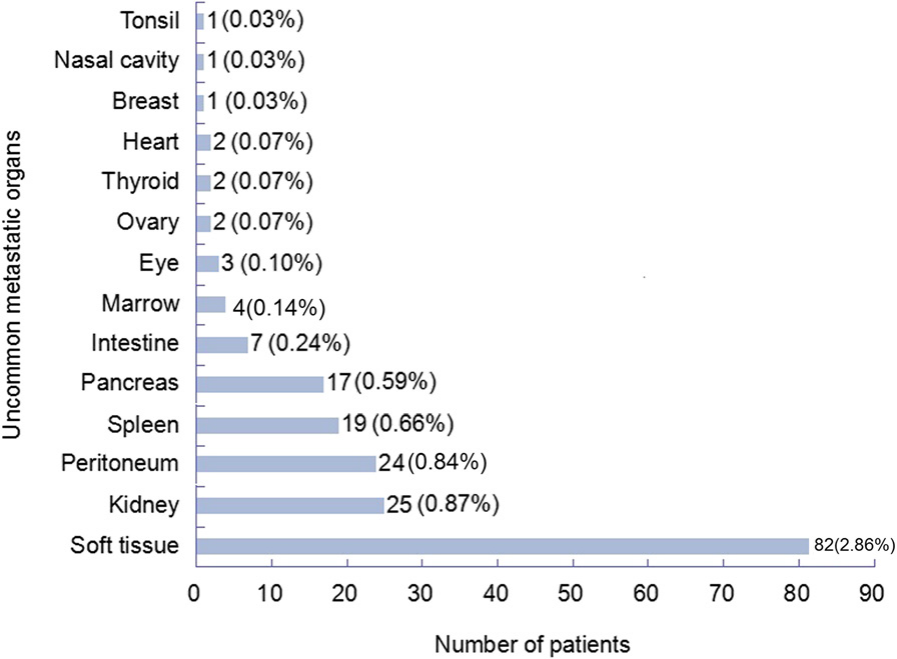
The frequency of uncommon metastases

The most uncommon metastatic sites, in decreasing order of frequency, were the soft tissue, kidney, peritoneum, spleen, pancreas, intestine, bone marrow, eye, ovary, thyroid, heart, breast, nasal cavity and tonsil. Figure 1 shows the frequency of metastasis at each uncommon site.

In total, 111 cases were diagnosed with synchronous metastases and 82 with metachronous metastases. In the latter group, the median duration between the initial diagnosis of lung cancer and the identification of uncommon metastases was 9.5 months. There were no significant differences in the other patient characteristics, including age, gender, smoking status, ECOG PS, histology and single-lesion or multiple-lesion metastases, between the two groups.

### Diagnosis and treatment

There were 84 uncommon metastatic sites diagnosed by pathology, 41 by PET/CT, 68 by CT, 14 by clinical signs, 4 by MRI. In the 84 uncommon metastases diagnosed by pathology, 71 patients were diagnosed with adenocarcinoma, 13 with squamous carcinoma, 2 with adenosquamous carcinoma, and 1 with squamous cell carcinoma combined with giant cell carcinoma. The histological features of the metastases were consistent with those of the primary tumors. Overall, 64 of the 84 patients had soft tissue metastases, 9 had peritoneal metastases, 3 had intestinal metastases, 1 had bone marrow metastases, and 1 each had kidney, pancreas, ovary, heart, breast, nasal cavity, and tonsil metastases.

In uncommon metastasis group, there were 157 patients received systematic treatment and the other 36 patients just received best supportive care (BSC) because of poor PS or poor financial condition. In common metastasis group, there were 2205 patients received systematic treatment and the other 474 patients just received BSC. The detailed treatment of both groups was shown in table 1.

After the diagnosis of uncommon metastases, 112 cases received systemic treatment without local treatment, 19 received systemic treatment plus local treatment, and the remaining patients received only BSC. Twelve and seven cases, respectively, in the synchronous and metachronous metastasis groups received local treatment. The systemic treatments included chemotherapy and targeted therapy. The local treatments included surgical extirpation, stereotactic body radiation therapy, and radiofrequency ablation.

### Survival analysis

A total of 151 cases with uncommon metastases died of lung cancer. The median OS (mOS) from the initial diagnosis of lung cancer to death was 13.0 months (95 % CI: 10.1–15.9 months) in 193 cases. The mOS from the diagnosis of uncommon metastases to death was 5.9 months (95 % CI: 4.6–7.0 months) in 193 cases.

**Fig. 2:**
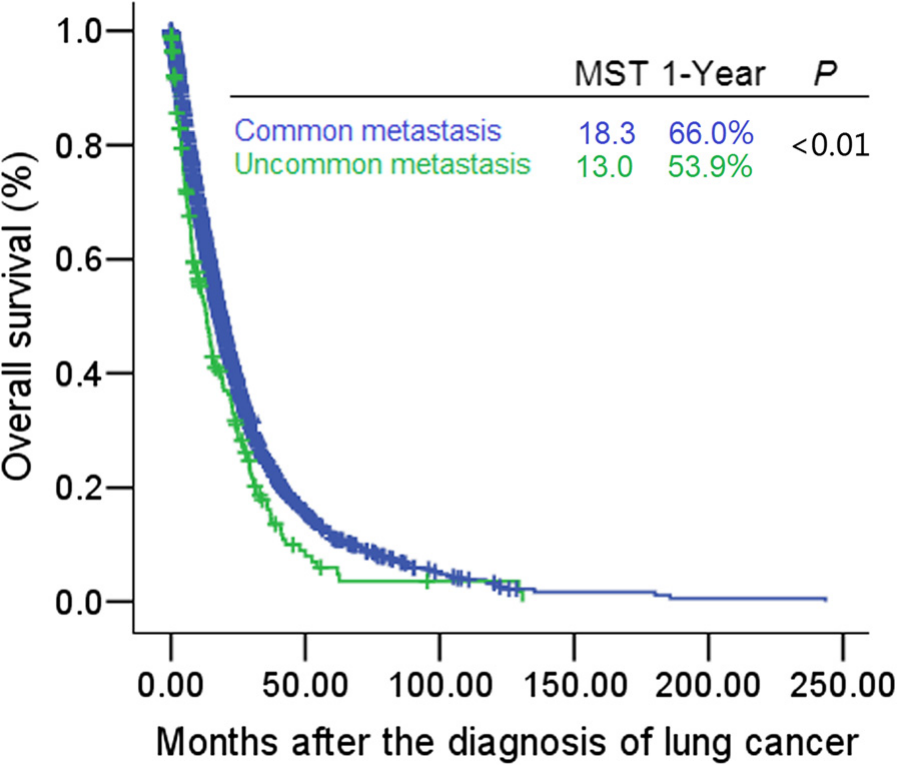
Survival from the time of lung cancer diagnosis in cases with common and uncommon metastases

**Fig. 3:**
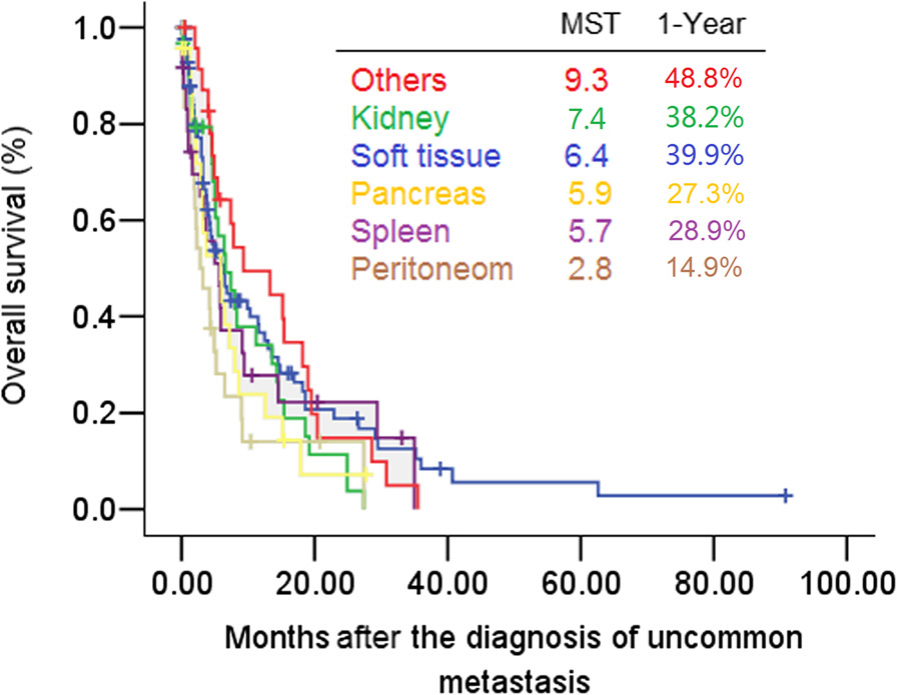
Survival from the time of the uncommon metastasis diagnosis. *: uncommon metastases except kidney/soft tissue/pancreas/spleen/peritoneum

As seen in Fig. 2, the mOS after the diagnosis of lung cancer was significantly shorter in patients with uncommon metastases versus common metastases (mOS 13.0 months [95 % CI: 10.1–15.9] *vs.* 18.3 months [17.4–19.2], *P* < 0.01). The 1-year survival rates were 53.9 and 66.0 %, respectively. Figure 3 shows the mOS after the diagnosis of uncommon metastases.

There was no significant difference in survival time after the diagnosis of uncommon metastases between the metachronous and synchronous metastasis groups (mOS 5.5 months [95 % CI: 3.4–7.6] *vs.* 6.0 months [95 % CI: 4.0–8.0], *P* = 0.91).

**Fig. 4:**
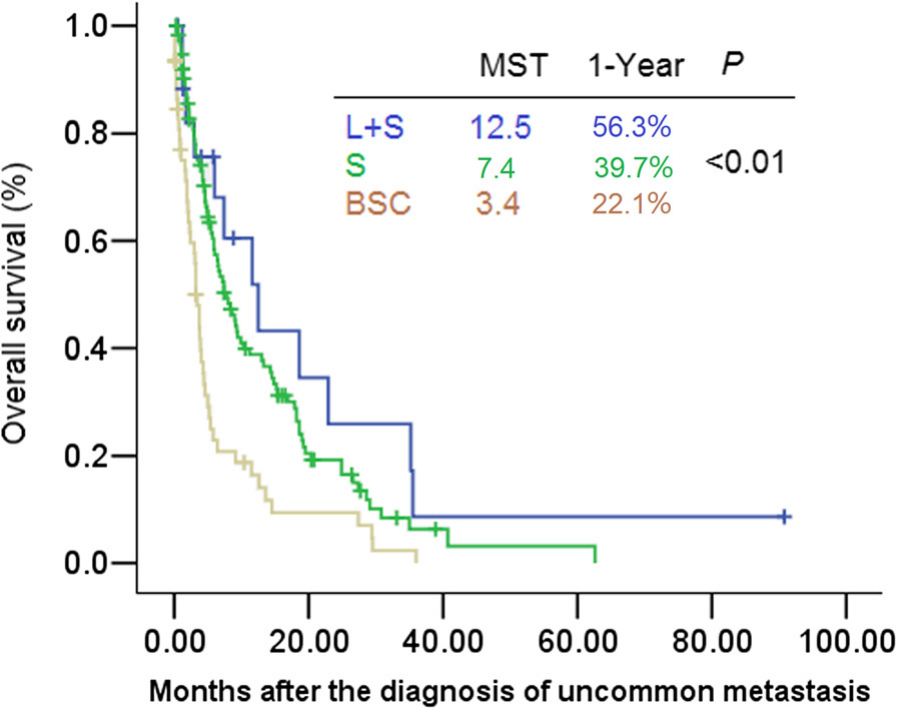
Survival from the time of the uncommon metastasis diagnosis in patients who received different treatments. Abbreviations: S, systemic treatment; L, local treatment; B, best supportive care

OS was significantly longer among patients who received systemic therapy plus local treatment compared to those who received systemic therapy alone, and those who received BSC (mOS: 12.5 months [95 % CI: 4.5–20.5] *vs.* 7.4 months [95 % CI: 5.2–9.6] *vs*. 3.4 months [95 % CI: 2.7–4.1]; *P* < 0.01) (Fig. 4).

### Multivariate stepwise Cox regression analysis

Four of the seven variables mentioned in the “patients and methods” section were found to be independent poor prognostic factors through a multivariate stepwise Cox regression analysis, as shown in Table 2. Uncommon metastases were associated with a hazard ratio (HR) of 1.29, following PS (HR = 2.15), no systematic treatment (HR = 2.06) and female (HR = 1.30).

**Table 2:** Independent prognostic factors from multivariate analysis between common and uncommon metastasis groups

	Harzard ratio	95 % CI	*P*
PS			
0–1	1.00	-	-
2	1.63	1.38–1.93	<0.01
>2	2.13	1.68–2.69	<0.01
Systematic treatment			
Yes	1.00	-	-
No	2.06	1.82–2.32	<0.01
Sex			
Female	1.00	-	-
Male	1.30	1.18–1.42	<0.01
Groups			
Common metastasis	1.00	-	-
Uncommon metastasis	1.29	1.09–1.52	<0.01

*Abbreviations*: *95 % CI* 95 % confidential interval, *PS* performance status

## Discussion

Numerous case reports and a few studies (with small sample sizes) that have evaluated uncommon metastases from lung carcinomas. Unfortunately, these reports have only marginally improved our understanding of the clinical features and outcomes of patients with the disease. Therefore, we reviewed the medical records of patients with NSCLC treated at the GLCI from 2006 to 2012 and reported the cases with uncommon metastases herein. To our knowledge, this is the largest case series of uncommon metastases from NSCLC.

As shown in Table 1, there were no significant differences in clinical characteristics at baseline between the common and uncommon groups except gender and diagnosis time. The predominant gender was male in cases with uncommon metastasis (*P* = 0.02), as has been noted in other reports [14]. Uncommon metastases were more likely to be diagnosed later in the disease course (*P* < 0.01). The reason why uncommon metastases most often occurs at disease course may be that the uncommon metastatic organs couldn’t provide suitable microenvironment to support tumor cells’ survival. It will take time for tumor cells to evolve to adapt the hostile microenvironment. The organs to which lung cancer uncommonly metastasizes are also usually unaffected in patients with other cancers. The reasons why these organs are rarely involved in cancers including NSCLC are as follows. K. A. Kovács, et al. [15] proposed that the skin only shared 5 % of the cardiac output of blood at resting condition, and was a very active part of immune system. The rarity of skeletal muscle metastases has been attributed to changes in pH, metabolite accumulation, variable blood flow, and variation in tissue pressure [16–18]. The spleen has always been considered “poor soil” for tumor growth, probably because of the high population of immune cells therein and the role played by that organ in “immune surveillance” [19]. Further, anti-angiogenesis factors produced by the spleen may explain the rarity of splenic metastases.

Uncommon metastases from lung tumors are included rarely in prognostic analyses. We speculated that patients with uncommon metastases would be more likely to have a poor outcome, according to previous case reports. A few articles have suggested that certain uncommon metastases, such as subcutaneous metastases [20], are associated with a shorter survival time. According to our multivariate analysis data, uncommon metastases independently predict a poorer survival on multivariate analysis (HR 1.29, *P* < 0.01). The fact that tumor cells could survive in hostile microenvironment provided by the uncommon organ (s) is evidence that such cells are particularly aggressive and invasive, which may contribute to the poor prognosis of these patients.

Uncommon metastases often develop late in the course of disease but also may present at initial diagnosis. We explored whether synchronous and metachronous metastases are different diseases or simply reflect different courses of the same disease [20]. We compared the baseline clinical characteristics and prognoses of the two groups, and found no significant difference in clinical characteristics or survival time after the diagnosis of an uncommon metastasis between the groups. It is possible that the patients in different groups represent different courses of the disease. Nonetheless, we couldn’t have a definitive explanation for this phenomenon without complete data for further analysis.

Uncommon metastases usually occur in patients with other disseminated metastases. Traditionally, systemic therapy is recommended as the standard therapy for metastatic disease. We administered local treatment in addition to systemic treatment to selected patients with oligometastases. More cases in the disease course group received local treatment. The patients who received systemic treatment plus local treatment had a better outcome than those who did not. Similar results have been observed in other studies [21, 22]. Therefore, we advise systemic treatment plus local treatment for selected patients with oligometastases.

A limitation of our study is its retrospective design, which spanned a long period of time. Regardless, it is the largest case series of uncommon metastases derived from NSCLC to date, and may aid clinicians in their treatment efforts.

## Conclusions

In conclusion, uncommon metastases are infrequently and associated with a worse outcome. Systemic treatment plus local treatment is a favorable option for selected patients with oligometastases.

## Abbreviations

MST: median survival time.
BSC: best supportive care
CI: confidence interval
CT: computed tomography
ECOG PS: Eastern Cooperative Oncology Group performance status
HR: hazard ratio
mOS: median overall survival
MRI: magnetic resonance imaging
NSCLC: non-small cell lung cancer
OS: overall survival
PET/CT: positron emission tomography/computed tomography
